# Machine learning-based neddylation landscape indicates different prognosis and immune microenvironment in endometrial cancer

**DOI:** 10.3389/fonc.2023.1084523

**Published:** 2023-02-22

**Authors:** Yi Li, Jiang-Hua Niu, Yan Wang

**Affiliations:** Department of Gynecology, The Affiliated Jiangsu Shengze Hospital of Nanjing Medical University & Jiangsu Shengze Hospital, Suzhou, China

**Keywords:** endometrial cancer, TME, prognosis, neddylation, machine learning

## Abstract

Endometrial cancer (EC) is women’s fourth most common malignant tumor. Neddylation plays a significant role in many diseases; however, the effect of neddylation and neddylation-related genes (NRGs) on EC is rarely reported. In this study, we first used MLN4924 to affect the activation of neddylation in different cell lines (Ishikawa and HEC-1-A) and determined the critical role of neddylation-related pathways for EC progression. Subsequently, we screened 17 prognostic NRGs based on expression files of the TCGA-UCEC cohort. Based on unsupervised consensus clustering analysis, patients with EC were classified into two neddylation patterns (C1 and C2). In terms of prognosis, substantial differences were observed between the two patterns. Compared with C2, C1 exhibited low levels of immune infiltration and promoted tumor progression. More importantly, based on the expression of 17 prognostic NRGs, we transformed nine machine-learning algorithms into 89 combinations. The random forest (RSF) was selected to construct the neddylation-related risk score according to the average C-index of different cohorts. Notably, our score had important clinical implications for EC. Patients with high scores have poor prognoses and a cold tumor state. In conclusion, neddylation-related patterns and scores can distinguish tumor microenvironment (TME) and prognosis and guide personalized treatment in patients with EC.

## Introduction

Endometrial cancer (EC) is women’s fourth most common malignant tumor. The incidence of EC in 2020 was 417,336, worldwide, and most EC cases occur between 65 and 75 years of age (1, 2). Especially, in developed countries, the incidence rate is the first among the three malignant tumors of the female reproductive system (3). When the metastasizes from the uterus, the survival rate drops dramatically, with 69% for patients with local metastases and only 17% for patients with distant metastases (4). Therefore, finding new therapeutic targets and developing agents with higher selectivity profiles for EC patients is urgent.

Post-translational modification (PTM) of proteins refers to the chemical modifications that proteins receive after RNA translation (5). PTM plays a crucial role in regulating protein conformation and function. To date, ubiquitination, SUMOylation, and phosphorylation have been shown to contribute to the EC progression of EC. Recent studies have shown that neddylation plays a crucial role in the development and progression of various cancers (6), including lung cancer (7), liver cancer (8), and prostate cancer (9). In these cancers, deregulation of the neddylation pathway can lead to an increase in cell proliferation and survival, leading to the development and progression of cancer. For example, neddylation has been shown to play a role in regulating the activity of the tumor suppressor protein p53, which is often mutated in cancers (10). Neddylation is the specific modification of ubiquitin-like small molecule NEDD8 by covalently binding lysine residues of substrate proteins in a multi-step enzymatic cascade reaction. The expression levels of NAE1, UBA3, UBC12, and UBE2F, critical enzymes involved in neddylation, and the level of neddylation in total proteins, are significantly increased in various tumors (11). However, the effect and mechanism of neddylation on EC are rarely reported. Significantly, most of the existing references focus on the mechanism and role of a specific gene in neddylation modifications. Therefore, it is relevant to systematically investigate the role of neddylation-related genes (NRGs) in predicting prognosis and tumor microenvironment (TME) in EC.

In our study, we first determined that the proliferation of EC cells (Ishikawa and HEC-1-A) was affected when neddylation modifications were inhibited using MLN4924. Subsequently, NRGs were collected from the Reactome database, and differences in the biological functions of different neddylation patterns in EC patients were determined. Ultimately, based on nearly 100 machine learning algorithms, a random forest tree (RSF) algorithm was determined to identify patient prognosis and TME based on the expression of NRGs in bulk RNA-seq.

## Materials and methods

### Datasets and data preprocessing

The TCGA-UCEC (TPM format) was downloaded from the TCGA database (12), in which a total of 587 samples contained RNA-seq data (35 normal and 552 tumor cases). Similarly, mutation data and copy number variation (CNV) data were downloaded from TCGA-UCEC. Among the 552 tumor samples, 9 cases of duplicate sequencing of the same sample were excluded, leaving 543 cases. Samples with no follow-up information for overall survival (OS) of less than one day were excluded. Finally, 541 tumor samples were included, which were subsequently divided into training and test sets in a 7:3 ratio using the “caret” package (version 6.0.92). The expression data were log2 transformed and normalized. The GeneMANIA database (13) was used to construct protein-protein interactions (PPI) network, and neddylation-related genes (NRGs) were obtained from the Reactome database (14) and previous references (15). Prognostic NRGs were selected for the following study based on the following criteria: p < 0.05.

### Cell proliferation assay

Human endometrial cancer cell lines Ishikawa and HEC-1-A were purchased from ATCC and cultured in 10% Gibco fetal bovine serum and 1% penicillin-streptomycin solution at 37°C with 5% CO2. MLN4924 powder was diluted with DMSO to prepare a 10mM storage solution for storage. The medium containing 0μM, 0.25μM, 0.5μM, 1.0μM MLN4924 was configured. The Ishikawa and HEC-1-A cells were seeded in triplicate into 96-well plates at 1×103 cells/well, then treated with different medium concentrations. Cell proliferation was determined by Cell Counting Kit-8 assay (Dojindo, Kumamoto, Japan) and ATP-Lite kit (PerkinElmer, Turku, Finland) according to the manufacturer’s instructions.

### Unsupervised consensus clustering

We used the “ConsensusClusterPlus” package (version 1.58.0) (16) to perform an unsupervised consensus clustering of the expression profile of prognostic NRGs, with the optimal number of clusters based on the cumulative distribution curve and K-means. The validity of the clustering was further confirmed by principal component analysis (PCA).

### Analysis of tumor immune microenvironment

Following the pipeline developed in previous studies (17), we used six algorithms (TIMER, CIBERSORT, QUANTISEQ, MCP-counter, XCELL, and EPIC) based “IOBR” package (version 0.99.9) to estimate the abundances of immune cells in different risk groups. In addition, we used ESTIMATE and ssGSEA to explore the TME status in different neddylation patterns (overall TME and 24 immune cell types).

### Functional enrichment analysis

Based on the Kyoto Encyclopedia of Genes and Genomes (KEGG)-related gene sets in the MSigDB (18), the enrichment score was calculated for each sample using “GSVA” package (version 1.42.0) (19) to obtain the enrichment score matrix. KEGG enrichment analysis was performed based on the differentially expressed genes (DEGs) between the two patterns (FDR < 0.05, |log2FC| > 1).

### Machine learning-derived prognostic neddylation-related risk score

We performed our previous workflow to construct a consensus prognosis model for EC patients (20, 21). Firstly, we constructed a combination of 89 machine learning algorithms based on the characteristics of the nine algorithms, including random forest (RSF), gradient boosting machine (GBM), survival support vector machine (Survival-SVM), supervised principal components (SuperPC), ridge regression, partial least squares regression for Cox (plsRcox), CoxBoost, Stepwise Cox (StepCox), and elastic network (Enet). Based on the previous workflow, we used models that can perform variable filtering as the antecedent models (RSF, CoxBoost, and StepCox). Subsequently, we used the training cohort from TCGA-UCEC and 89 combinations to construct signatures in an expression file with prognostic NRGs. Finally, the neddylation scores were calculated using the signatures obtained in the training cohort, both in the testing cohort and in all cohorts. We selected the best consensus prognostic model for EC based on the mean C-index of the three cohorts.

### Evaluating clinical significance of neddylation-related risk score

We compared the differences between high and low-risk groups on clinical characteristics such as age, FIGO stage, and grade. In addition, further Kaplan-Meier analysis was performed with clinical subgroups. Receiver-operator characteristic (ROC) curves were plotted for each cohort to assess the predictive accuracy of the risk score.

### Statistical analysis

All statistical analyses in genome were performed using the R software (v.4.1.2). More detailed statistical methods are covered in the above section (22, 23). The assessment of correlations between continuous variables was performed using Pearson’s correlation coefficients. For comparison of categorical variables, the chi-squared or Fisher exact test was applied, while the Wilcoxon rank-sum test or T test was utilized for comparison of continuous variables. P < 0.05 was considered statistically significant.

## Results

### MLN4924 suppresses proliferation of EC cells

**Figure 1:**
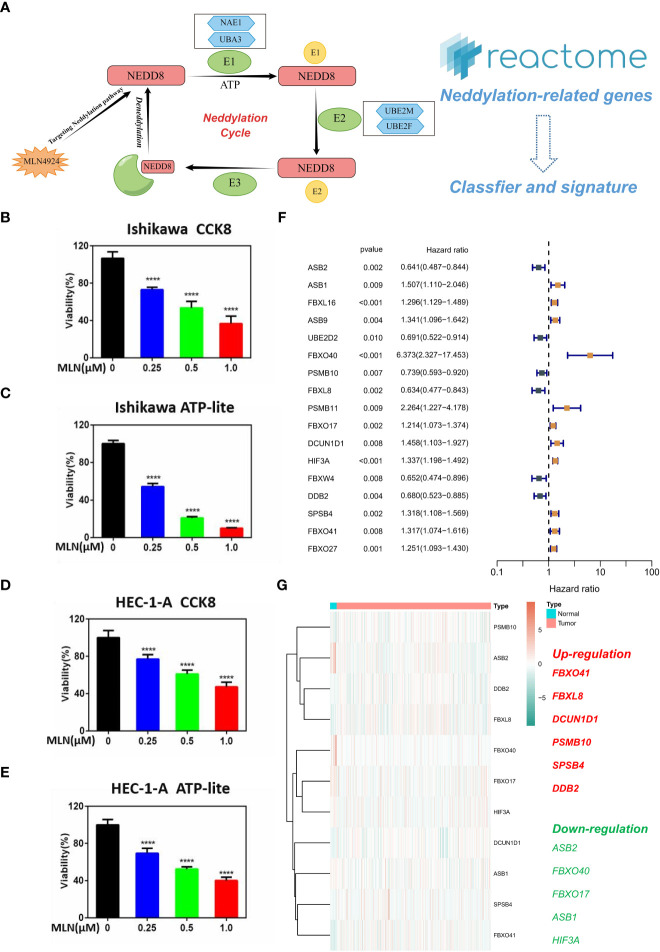
Key role of neddylation-related pathway in EC. **(A)** Process of neddylation modification. **(B, C)** MLN4924 significantly inhibited the proliferation of Ishikawa cell. **(D, E)** MLN4924 significantly inhibited the proliferation of HEC-1-A cell. **(F)** Univariate Cox analysis of neddylation-related genes in TCGA-UCEC cohort. **(G)** Expression landscape of 17 prognostic NRGs. ****p < 0.0001.

Neddylation is an important PTM; It is a reversible process regulated by NEDD8, NAE1, UBA3, UBE2M, UBE2F, and other neddylation-related proteins (**Figure 1A**). The activation of Cullin-RING E3 requires Neddylation modification of its core subunit Cullin, and blocking the Neddylation modification of Cullin can inactivate the CRL ubiquitin ligase function. Based on this key feature, the small molecule inhibitor MLN4924 was successfully developed by high-throughput drug screening (24). We inoculated Ishikawa and HEC-1-A in 96-well plates at appropriate cell densities and treated the cells with different concentrations of MLN4924 for 96 h on the following day. The cell viability was detected using the CCK8 and ATP-lite kit. The results showed that the cell viability of the MLN4924-treated group was lower than a the control group, and the cell viability decreased as the concentration of MLN4924 increased, indicating that MLN4924 significantly inhibited the proliferation of EC cells (**Figures 1B–E**). Subsequently, we used univariate Cox analysis on all NRGs in the Reactome database, resulting in the identification of 17 NRGs with significant prognostic value (**Figure 1F**). To further explore the expression of 17 NRGs in EC, we performed a differential analysis in the TCGA-UCEC cohort (**Figure 1G**). The results showed that FBXO41, FBXL8, DCUN1D1, PSMB10, SPSB4 and DDB2 was up-regulated in tumors and, in contrast, ASB2, FBXO40, FBXO17, ASB1 and HIF3A was down-regulated. These results demonstrate an essential role of neddylation modification for EC progression.

### Genetic variation of 17 prognostic neddylation-related genes in EC

**Figure 2:**
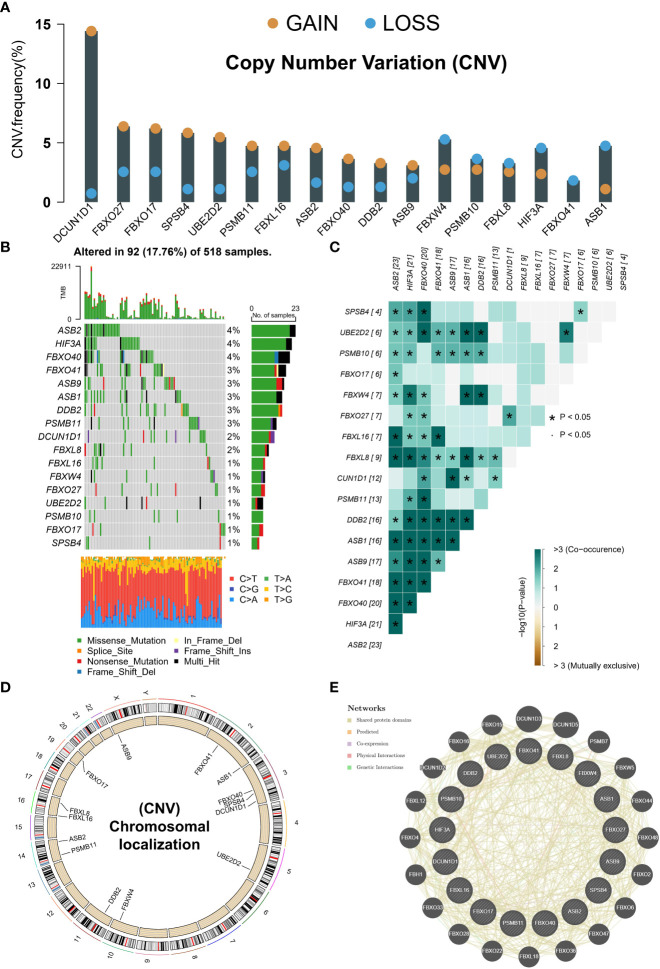
Genetic variants in prognostic NRGs. **(A)** CNV in prognostic NRGs. **(B)** Mutation landscape of prognostic NRGs. **(C)** Co-mutation landscape of prognostic NRGs. **(D)** The location of a prognostic gene on chromosome. **(E)** PPI network of prognostic NRGs in GeneMANIA database. *p < 0.05.

CNV amplification was prevalent in many NRGs, especially DCUN1D1 and FBXO27. At the same time, CNV deletion was mainly found in ASB1, HIF3A, and FBXW4 (**Figure 2A**). The TCGA-UCEC cohort contains 518 exons sequenced samples. A total of 17.76% of mutation frequencies were found in these NRGs, mainly manifesting as missense mutations (**Figure 2B**). Interestingly, there was a significant co-mutation relationship in prognostic NRGs (**Figure 2C**). CNV occurs on many chromosomes but was mainly concentrated on chromosomes 3 (**Figure 2D**). Finally, we constructed a PPI network of NRGs, notably the PPI network mainly consisted of shared protein domains that exert interactions (**Figure 2E**). These results suggest that prognostic neddylation-related genes have interactions and may play synergistic roles at the protein level.

### Identification of different neddylation patterns of EC

**Figure 3:**
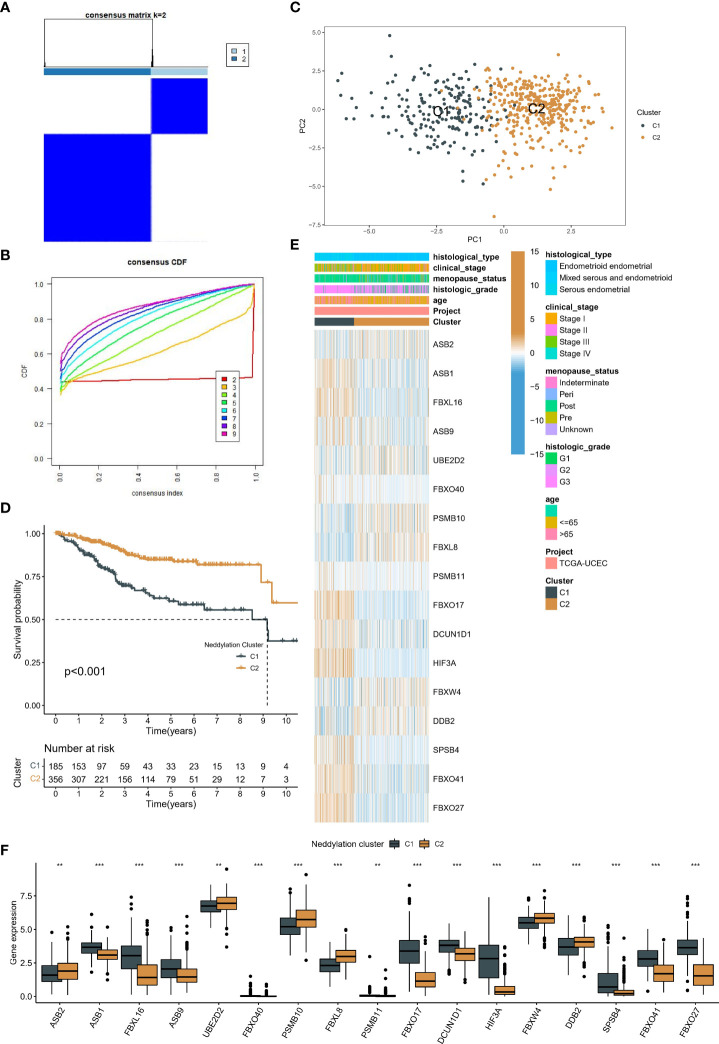
Identification of two patterns associated with neddylation in EC patients. **(A, B)** The optimal number of clusters based on the cumulative distribution curve. **(C)** PCA in two patterns. **(D)** Kaplan-Meier analysis of prognosis between the two patterns. **(E-F)** Differences in clinical characteristics and prognostic NRGs expression between the two patterns. **p < 0.01, ***p < 0.001.

Based on the consensus clustering algorithm, the best clustering was achieved when k=2 (**Figures 3A, B**), so we classified TCGA-UCEC into two neddylation-related molecular subtypes, C1 (n=185) and C2 (n=356). Where PCA illustrated that different subtypes were heterogeneous and may represent levels of neddylation modifications (**Figure 3C**). Importantly, C1 showed a worse prognosis than C2 (**Figure 3D**) in OS analysis. As with OS, molecular typing has the same prognostic value in disease free survival (DFS) (**Supplementary Figure 1**). Subsequently, we heat mapped the clinicopathological factors and 17 prognostic NRGs of these two subtypes in EC patients (**Figures 3E, F**). Interestingly, there were differences in the expression of all prognostic NRGs between the two subtypes, and C2 with a better prognosis had more patients in G1 and stage I (**Figure 3G**). These results support the theory that different neddylation patterns represent different prognoses and clinical characteristics.

### TME and functional differences between the two neddylation patterns

**Figure 4:**
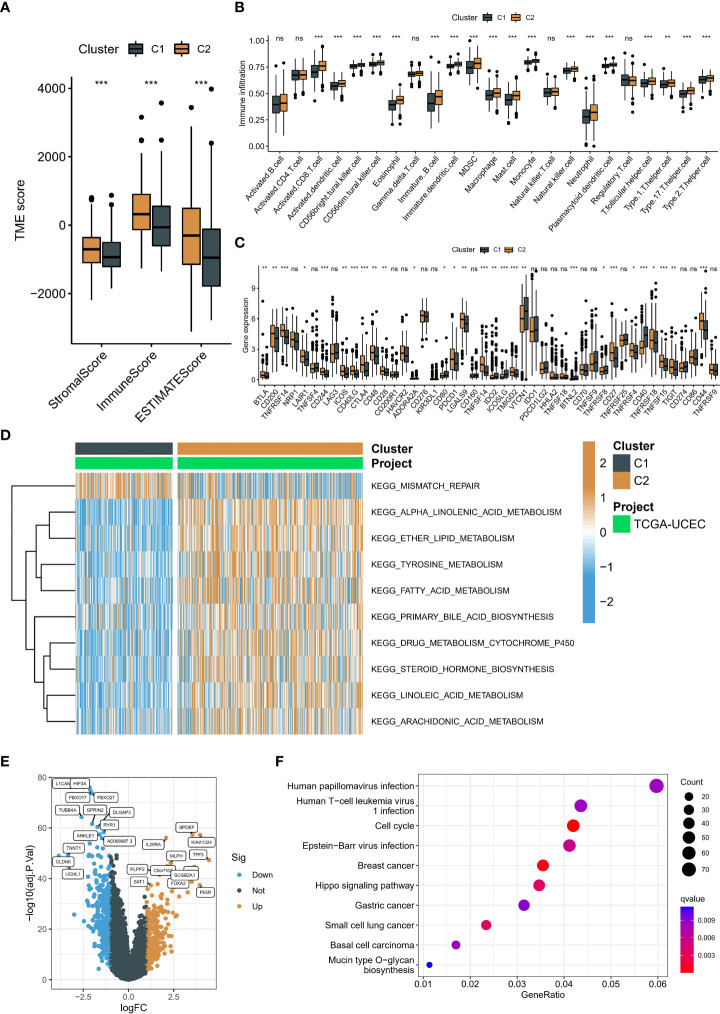
Immune landscape and biological function in two patterns associated with neddylation. **(A)** Overall TME status between the two patterns based on ESTIMATE algorithm. **(B)** Abundance of 24 immune cells based on ssGSEA algorithm. **(C)** Differences in mRNA associated with immune checkpoint inhibitors. **(D)** Heatmap of matrix of KEGG enrichment scores using GSVA algorithm. **(E)** Volcano map showed DEGs between two patterns. **(F)** KEGG enrichment analysis of DEGs. ns, no statistical significance; *p < 0.05, **p < 0.01, ***p < 0.001.

To investigate the causes of the different prognosis in two neddylation patterns, we used the ESTIMATE algorithm to assess the TME landscape of TCGA-UCEC samples. We found that C1 had a higher immune and stromal score (**Figure 4A**). In addition, these results were further validated by using ssGSEA, and C2 typically had a lower abundance of immune cells, such as B cells, T cells, NK cells (**Figure 4B**). In short, both algorithms suggested that C2 had a lower level of immune infiltration, which may facilitate tumor escape. These findings were consistent with the prognostic results in the above section. More importantly, we compared the differences in immune checkpoint expression levels (mRNA) in two neddylation patterns. Interestingly, C2 had higher expression levels of most immune checkpoints, such as PDCD1, CTLA compared with C1, suggesting that patients in C1 may benefit from immunotherapy (**Figure 4C**). Interestingly, patients with poor prognosis had lower Myeloid-derived suppressor cells (MDSCs) levels and lower mRNA expression of Programmed cell death protein 1 (PD-1) compared with C2. Moreover, GSVA was used to calculate the scores of gene sets from the KEGG pathway and ultimately found that only the mismatch repair pathway was significantly enriched in C1. In contrast, all other pathways were enriched in C2, such as alpha-linolenic acid metabolism, primary bile acid biosynthesis (**Figure 4D**). Finally, we performed KEGG enrichment analysis based on the differential expression genes (DEGs) of the two neddylation patterns (**Figure 4E**), with results of significant enrichment of cell cycle and Hippo signaling pathways (**Figure 4F**). Taken together, these results of the ssGSEA and GSVA algorithms showed that C1 exhibits a pro-progression pattern in molecular mechanisms, which explains the poorer prognosis for C1.

### Integrated development of a neddylation-related signature in EC patients

**Figure 5:**
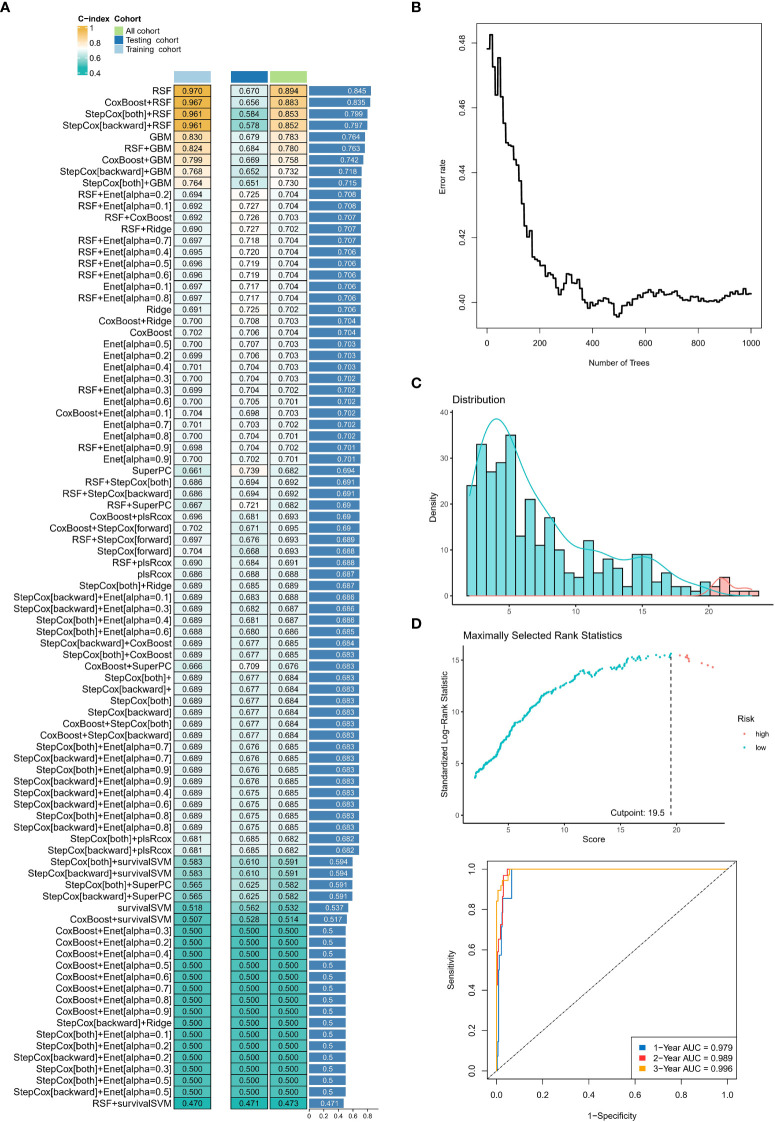
Construction and testing of the machine learning-derived prognostic signature. **(A)** The C-indexes of 89 machine-learning algorithm combinations in the three cohorts. **(B)** Error rate curve of random forest tree model. **(C)** The changes in AUC value of different time points. **(D)** The area under the ROC curve (AUC) for the 1-year, 2-year, and 3-year OS in the training cohort. ns, no statistical significance; *p < 0.05, **p < 0.01, ***p < 0.001.

We calculated the average C-index for each algorithm in all cohorts and selected the RSF algorithm with the highest average C-index (0.845) as the final model (**Figure 5A**). We further calculated the risk score for each sample in the cohort based on the expression file of the 13 NRGs included in the RSF model (HIF3A, ASB2, ASB1, FBXO27, SPSB4, UBE2D2, DDB2, FBXO41, FBXL8, FBXW4, FBXO17, FBXL16, and ASB9) (**Figure 5B**). Subsequently, we determined the optimal cut-off value (19.5) based on the risk scores in the training set (**Figure 5C**). Notably, the area under the ROC curve (AUC) for the 1-year, 2-year, and 3-year OS was 0.979, 0.989, and 0.996 in the training cohort (**Figure 5D**).

### Robust predictive performance of neddylation-related signature

**Figure 6:**
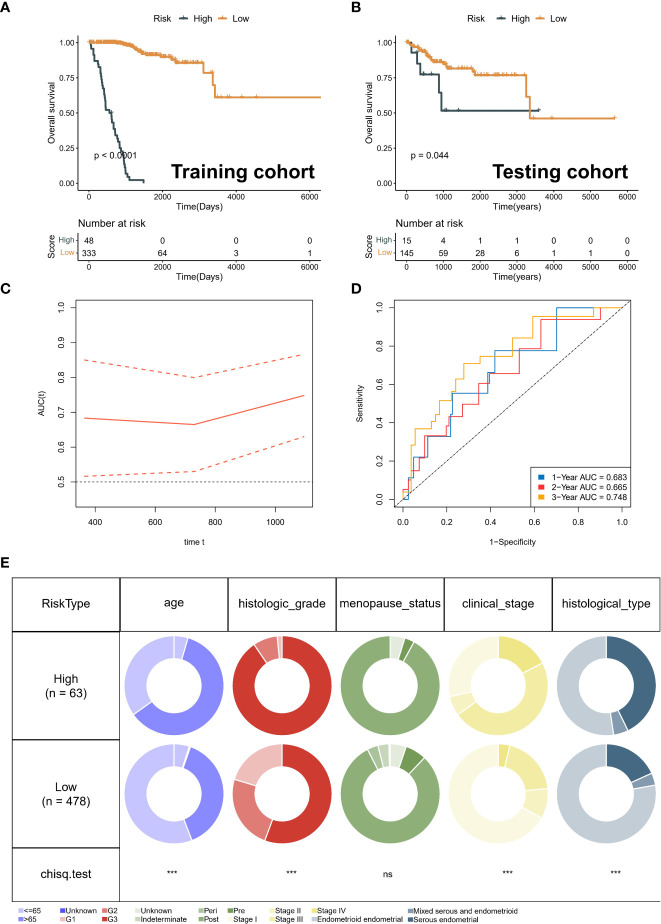
Validation of prognostic signature. **(A)** Kaplan-Meier analysis of different risk group in training cohort. **(B)** Kaplan-Meier analysis of different risk group in testing cohort. **(C)** Risk score distribution map and optimal cut-off value selection. **(D)** The area under the ROC curve (AUC) for the 1-year, 2-year, and 3-year OS in the testing cohort. **(E)** The distribution between risk groups and clinical features. ns, no statistical significance.

**Figure 7:**
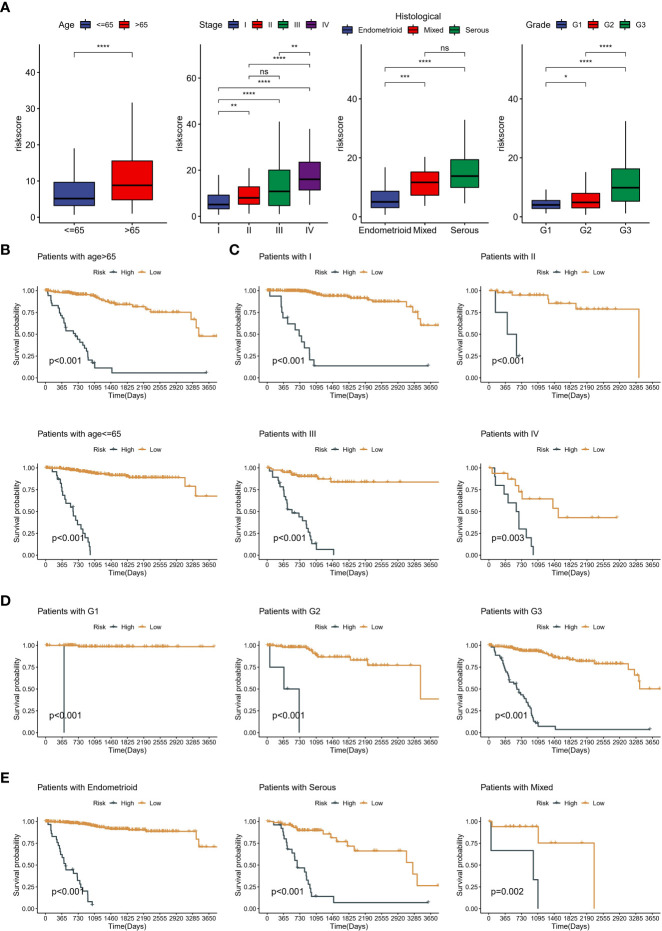
Clinical significance of neddylation-related risk score. **(A)** The differences in risk scores across clinical subgroups, including age, grade, FIGO stage and pathologic types. **(B)** Kaplan-Meier analysis of different age subgroups. **(C)** Kaplan-Meier analysis of different FIGO stage subgroups. **(D)** Kaplan-Meier analysis of different grade subgroups. **(E)** Kaplan-Meier analysis of different pathologic types subgroups. ns, no statistical significance; *p < 0.05, **p < 0.01, ***p < 0.001, ****p < 0.0001.

Kaplan-Meier curves for OS showed that the low-risk group significantly had more prolonged survival in the training cohort (**Figure 6A**). The testing cohort also showed the same trend (**Figure 6B**). As with OS, risk typing has the same prognostic value in disease free survival (DFS) (**Supplementary Figures 2A-B**). Moreover, we showed the changes in AUC. We found that the AUC at different time points in the testing cohort is all around 0.7 (**Figure 6C**). The AUC for the 1-year, 2-year, and 3-year OS was 0.683, 0.665, and 0.748 in the testing cohort (**Figure 6D**). Importantly, the distribution of high and low-risk group was significantly different in age, grade, stage and pathological type (**Figure 6E**). Subsequently, we grouped all patients by different clinical characteristics. We found that the risk score increased significantly with increasing age, higher FIGO stage, higher grade, and poorer pathological types (serous) (**Figure 7A**). Surprisingly, risk scores also had a significant ability to differentiate survival status in different clinical subgroups (**Figures 7B–E**).

### Genome alteration landscape and TME status of neddylation-related signature

**Figure 8:**
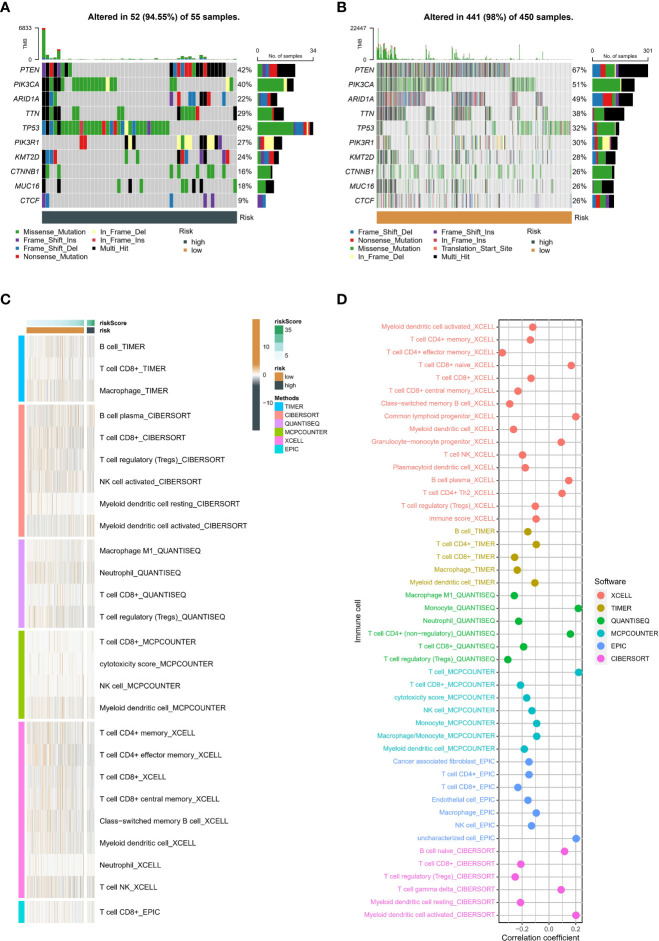
Genetic variants and tumor microenvironment in different risk. **(A)** The top 10 mutated genes in the high-risk group. **(B)** The top 10 mutated genes in the low-risk group. **(C)** Difference of abundance of immune cells based on six algorithm in different risk groups. **(D)** Correlation between abundance of immune cells based on six algorithm and risk score.

The heatmap identified the ten genes with the highest mutation rates in different groups (**Figures 8A, B**), such as PTEN, PIK3CA, ARID1A, and TTN. The above ssGSEA suggested that TME status differs in different neddylation patterns. Hence, we also used six algorithms to explore the differences in the TME status in different risk groups (**Figure 8C**). We found that the low-risk group exhibited a relatively high abundance of infiltrated immune cell types, including activated CD4^+^ T cells, activated CD8^+^ T cells, etc. More importantly, the correlation analysis likewise showed that as the risk score increased, the immune cell abundance score decreased (**Figure 8D**).

## Discussion

Neddylation modification is a novel protein post-translational modification recently reported as an ATP-dependent biological enzymatic cascade reaction, which involves the covalent modification of NEDD8 to bind to the substrate target protein catalyzed by NEDD8 activating enzyme E1, NEDD8 binding enzyme E2 and a few NEDD8 ligases E3 in succession (25), thereby regulating the conformation and activity of the target protein. In this study, we found that the Neddylation modification pathway is activated in EC cells and in the first section, we have explored the mechanism of MLN4924 affects EC cells, which provides a scientific basis for identifying MLN4924 as a new anti-EC drug. Based on this evidence, further exploration of neddylation-related tumor classification and risk stratification may be possible to determine the prognosis of EC and provide personalized treatment options.

Firstly, we used univariate Cox analysis on all NRGs, identifying 17 NRGs with significant prognostic value. We classified TCGA-UCEC into two neddylation-related patterns based on the consensus clustering algorithm. Interestingly, there were differences in the expression of all prognostic NRGs between the two patterns, and C2 with a better prognosis, had more patients in G1 and stage I. The results of ssGSEA further suggested that C2 typically had a lower abundance of immune cells, such as B cells, T cells, and NK cells. This is consistent with current research, where neddylation has also been critical in various processes in the human immune system, including inflammation, viral infection, and regulation of T-cell function (26). MLN4924 inhibits NEDD8-activating enzyme and then regulates the H1 phenotype of T cell polarization registration differential and shift to T in chronic lymphocytic leukemia patients with lower T, but increases IFN-γ (27). Endogenous acylated Cul-4b are more abundant after T cell activation and are required to maintain effector CD4^+^ T cell viability (28). Moreover, MLN4924 leads to impaired NEDD8-dependent clearance of misfolded proteins and an altered tumor immune microenvironment due to increased numbers of cytotoxic T cells and conventional CD4^+^ T cells and decreased numbers of regulatory T cells (29). Interestingly, in the C1 patients with poor prognosis, lower abundance of MDSCs and a lower expression of PD-1 suggest that the immune system is less able to suppress T-cell activity. However, from the perspective of the overall immune microenvironment, the ESTIMATE results suggest that CI subtypes are in the state of cold tumors. This may result in a more aggressive cancer and a poorer prognosis for the patient. However, further research is needed to fully understand the underlying biology and the relationship between MDSCs, PD-1, and EC prognosis.

Interestingly, GSVA was used to calculate the scores of gene sets from the KEGG pathway and ultimately found that only the mismatch repair (MMR) pathway was significantly enriched in C1. Loss of MMR function induces a hypermutation phenotype clinically recognized by a genomic scar called microsatellite instability (MSI) (30). Notably, the highest prevalence of MSI was in endometrial cancer (31.4%) (31). The prognosis of EC patients with MSI is similar to that of endometrioid patients (32). Interestingly, one study showed that after treatment with MLN4924, dMMR/MSI tumors accumulated misfolded proteins, thereby activating the unfolded protein response. This again demonstrates the importance of our identified neddylation pattern as a therapeutic guide.

Given that the neddylation patterns are not directly available for clinical use and the lack of biomarkers for prognostic follow-up, we used the expression of 17 prognostic NRGs to construct 14-NRGs prognostic signatures by combining a large number of machine learning algorithms. More importantly, our neddylation-related risk score exhibits powerful and superior predictive power. Notably, the AUC for the 1-year, 2-year, and 3-year OS was 0.979, 0.989, and 0.996 in the training cohort. Importantly, the distribution of high and low-risk group was significantly different in age, grade, stage and pathological type. We found that the risk score increased significantly with increasing age, higher FIGO stage, higher grade, and poorer pathological types (serous). Surprisingly, risk scores also had a significant ability to differentiate survival status in different clinical subgroups.

The present study differs from previous references in the following aspects. (1) The importance of the neddylation in EC was confirmed experimentally and followed by bioinformatic analysis. (2) We selected the algorithm with the most extensive mean C index across cohorts to construct our risk model. (3) To prevent inappropriate modeling approaches due to personal preferences, we combined machine learning algorithms into 89 combinations and selected the best model. However, some limitations should be noted in our study. First, there is no suitable external validation cohort other than the TCGA database. Second, 14 NRGs are related to neddylation, but their other roles in EC remain to be elucidated, and more experimental validation is needed in the future. Importantly, future perspectives of neddylation-related prognostic models include: Improved accuracy: Neddylation-related prognostic models will become more accurate and reliable as more data and insights are gained into the complex molecular mechanisms underlying neddylation and EC. Personalized medicine: our models can be used to predict the response of individual patients to different treatments and inform the development of personalized medicine strategies. Improved early detection: Neddylation-related genes can be used for early detection of EC and to monitor the progression of the disease, allowing for earlier and more effective interventions. Combination with other biomarkers: risk score can be combined with other biomarkers, such as CA125, CA199, CA153, to improve the accuracy and reliability of EC diagnosis and prognosis.

## Data availability statement

The datasets presented in this study can be found in online repositories. The names of the repository/repositories and accession number(s) can be found in the article/**Supplementary Materials**.

## Author contributions

YL conceived and designed the study. YL was responsible for materials. YL drafted the article. J-HN and YW revised the article critically. All authors contributed to the article and approved the submitted version.

## Supplementary material

Supplementary Figure 1 Kaplan-Meier analysis of DFS between the two patterns.

Supplementary Figure 2 Kaplan-Meier analysis of DFS in different cohorts. (A) Training cohort. (B) Testing cohort.
