# Supplementary figures and images for: Machine learning-based neddylation landscape indicates different prognosis and immune microenvironment in endometrial cancer
Source: Front Oncol. 2023 Feb 22;13:1084523. doi: 10.3389/fonc.2023.1084523 (PMC9992729; doi:10.3389/fonc.2023.1084523)

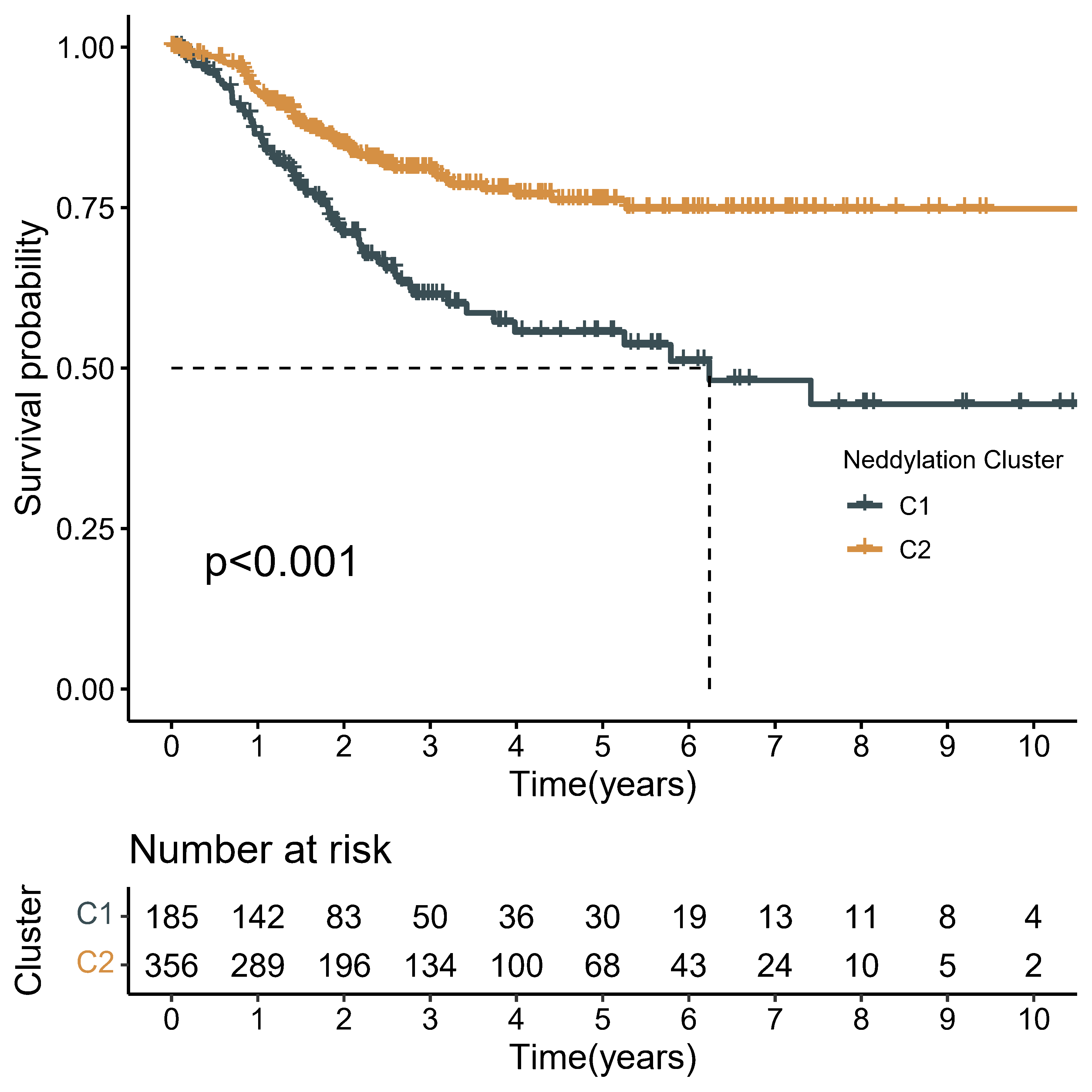

Supplement: Supplementary Figure 1 — Kaplan-Meier analysis of DFS between the two patterns. [file Image_1.tif]

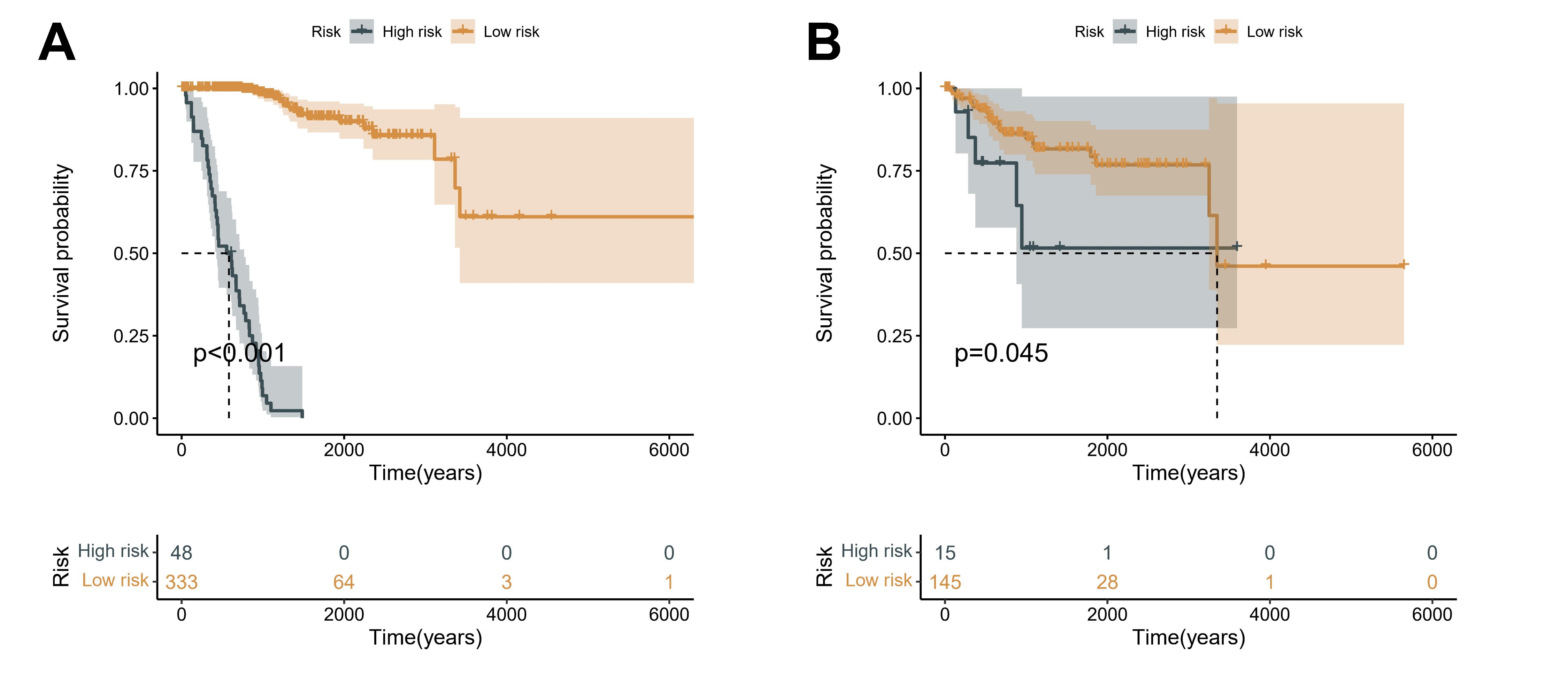

Supplement: Supplementary Figure 2 — Kaplan-Meier analysis of DFS in different cohorts. (A) Training cohort. (B) Testing cohort. [file Image_2.tif]
